# Comparison of QT dispersion before and after PDA device closure in pediatrics

**DOI:** 10.1111/anec.12945

**Published:** 2022-03-10

**Authors:** Mohammad Reza Khalilian, Mehdi Ziaratban, Parinaz Alizadeh, Ali Reza Norouzi, Armin Shirvani

**Affiliations:** ^1^ Department of Pediatrics School of Medicine Shahid Beheshti University of Medical Sciences Tehran Iran; ^2^ Pediatric Respiratory Diseases Research Center (PRDRC) National Research Institute of Tuberculosis and Lung Diseases (NRITLD) Masih Daneshvari Hospital Shahid Beheshti University of Medical Sciences Tehran Iran

**Keywords:** patent ductus arteriosus, pediatrics, QT dispersion

## Abstract

**Background:**

Numerous studies have shown that QT dispersion (QTd) can be a suitable criterion for risk assessment of arrhythmia in patients with congenital heart disease. Pulmonary arterial hypertension (PAH) increases the risk of cardiac arrhythmia by changing ventricular repolarization homogeneity. In this study, we assessed QTd changes after PDA device closure and the effect of PAH on these changes.

**Methods:**

Between October 2018 and March 2021, 97 patients (48 males; 49 females; mean age 31.36 ± 4.26 months; range 3 months to 14 years) who satisfied the primary inclusion criteria and did not meet the exclusion criteria and underwent PDA device closure intervention were included in the study. Echocardiography was performed before the procedure. QT corrected (QTc), and QTd and PR intervals were measured according to the patients’ standard 12‐lead ECGs in two periods, preoperative (1 day) and after (3 months).

**Results:**

In the general group, QTc and QTd decreased significantly after PDA closure. Based on our classification of the patients in two groups of high PAP and normal PAP, the three parameters QTc, QTd, and PR interval were assessed separately in the two groups. All three parameters decreased significantly in the normal PAP and high PAP groups.

**Conclusions:**

However, a left‐to‐right shunt through the patent ductus arteriosus can affect ventricular repolarization; this effect seems to be particularly more significant when there is pulmonary hypertension.

## Graphical abstract

This study was conducted on 97 pediatric patients with PDA who candidate for PDA device closure. The patients were divided into two normal and high PAP groups based on measurement of pulmonary pressure in cardiac catheterization (67 patients were identified as the normal PAP group and 30 patients as the high PAP group). ECG findings (PR interval, QT corrected, and QT dispersion) were assessed before and after PDA device closure.

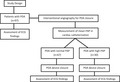

## 1 INTRODUCTION

For the first time, Mirvis noticed a significant irregularity of the QT intervals in patients with acute myocarditis compared to the healthy population (Mirvis, 1985). Later, in 1990, Day et al. proposed a potential application of this inter‐lead difference using standard 12‐lead electrocardiograms (ECGs) (Day et al., 1990). Since then, numerous studies have investigated the differences between QT max and min in different ECG leads, entitled QT dispersion (QTd) (Day et al., 1990). Several studies have shown an association between death from a cardiac cause and QTd (Barr et al., 1994; Bazoukis et al., 2020; Naas et al., 1998). It should be noted that some findings have challenged this association. For example, QTd may decrease as a result of drug consumption (Fei et al., 1996; Moreno et al., 1994). Also, an increase in QTd in patients with idiopathic dilated cardiomyopathy is not linked to the increase in mortality (Fei et al., 1996). However, in addition to these studies, it seems that QTd can be a suitable criterion for risk assessment of arrhythmia in patients with long QT, hypertrophic cardiomyopathy, and sustained ventricular arrhythmias (Day et al., 1990; Fei et al., 1996; Pye et al., 1994). As we know, pulmonary arterial hypertension (PAH) is a common complication of congenital heart disease (Farag et al., 2018). Pulmonary arterial hypertension increases the risk of cardiac arrhythmia by changing ventricular repolarization homogeneity in three ways, including modulating autonomic activity, delaying cardiac repolarization, and causing right ventricular myocardial ischemia (Rajdev et al., 2012; Temple, 2017). The importance of research on QTd and using the parameter is that it is completely non‐invasive and, unlike Holter monitoring, can be achieved from a single 12‐lead ECG within a short period (Linker et al., 1992; Zaidi, 1996). Prolonged dispersion in QT is associated with ventricular arrhythmias and sudden death in patients with PAH, coronary, and chronic heart disease (Barr et al., 1994; Blužaitè et al., 2006; Brendorp et al., 2001). A study by Saleh et al. regarding this relationship showed that QTd had 93% sensitivity, 80% specificity, and 85% accuracy in predicting the occurrence of arrhythmias in patients with PAH‐CHD (*p* = .003 and *p* = .01, respectively). The relationship was independent of patients’ age, sex, and weight (Saleh et al., 2019). Also, QTd was more prolonged in patients with PAH (Saleh et al., 2019). In a study by Ece et al. in 2014, QTd was higher in patients with Eisenmenger syndrome (*p *< .001) (Brendorp et al., 2001). Open interventions on the heart have shown to increase QT and QTd (*p* < .001) (Ece et al., 2014). QT and QTc decrease after the operation (*p *< .02) but finally increase after 4 months of open‐heart surgery compared to the pre‐operation period (*p *< .001). The increase in QTd on the day after surgery, which was associated with an increased risk of mortality, is significant, but seemingly, QTd returned to its previous level only 2 months after surgery (Alp et al., 2012).

This finding is inconsistent with the results of a study by Alp et al. in 2012, based on comparing ECGs of 279 children who underwent open‐heart surgery. It was shown that QTd increased significantly after surgery (Alp et al., 2012).

## 2 METHODS

### 2.1 Patients population

This prospective cross‐sectional study was conducted on 97 patients with PDA who were admitted to the Pediatric Cardiology Ward, between October 2018 and March 2021. The patients were further divided into two normal and high PAP groups based on measurement of pulmonary pressure in cardiac catheterization (30 patients were identified as the high PAP group and 67 patients as the normal PAP group) according to the Sixth Pulmonary Hypertension World Symposium 2018, PAH refers to the mean pulmonary artery pressure above 20 at rest. The inclusion criteria comprised infants and children diagnosed with PDA, with or without PAH, and any size PDA. Exclusion criteria included other structural cardiac anomalies and known risk factors affecting QT intervals such as drugs, electrolyte disturbances, heart blocks, bundle branch block, suspicion of metabolic or genetic disorders, and patients with a history of previous cardiac surgery.

### 2.2 Electrocardiography and other assessments

The study participants underwent a complete patient history‐taking and several clinical examinations, including heart rate monitoring, O_2_ saturation, and complete cardiac examination. The pre‐test 12‐lead surface ECGs of patients were used to measure PR and QTc intervals and QT dispersion. A single operator performed all the measurements manually. QT interval was measured from the beginning of QRS complex to the end of T wave in a single beat in all 12 leads. All QT intervals were corrected by RR interval, using Bazett formula (QTc =QT/√RR). PR interval and QT interval dispersions were measured manually by handheld calipers and magnification. QTd is defined by difference between maximum and minimum QT intervals in various 12‐lead ECG leads at rest. Chest X‐ray was carried out before PDA closure for measuring cardio‐thoracic ratio and pulmonary blood flow. Echocardiography was conducted using a vivid ultrasound machine to detect PDA size and pulmonary artery pressure.

### 2.3 Interventional angiography

Mean pulmonary artery pressure (MPA) was calculated based on cardiac catheterization. We divided our patients into two normal and high PAP groups (considering MPA>20 mmHg as high PAP). Patients’ PDA was closed by interventional angiography. Children with residual PDA after intervention were excluded in this study.

### 2.4 Patients follow‐up

Our primary objective was to assess QTd, QTc, and PR interval changes following PDA closure intervention. The secondary objective was to investigate the influence of pulmonary arterial pressure on QTd changes following the procedure. For this we performed an echocardiography 3 months after procedure for detecting residual PDA and a 12‐lead ECG for measuring QTd, QTc, and PR interval.

### 2.5 Statistical analysis

After gathering mentioned information, these data were entered into Excel software. Statistical analyses were performed by SPSS software version 23. Independent paired t test was used to evaluate the significance of changes in QTc, QTd, PR interval. Pairwise correlation coefficient test was used to evaluate the effect of pulmonary artery pressure on QTc, QTd, and PR interval changes. Regression analysis was used to evaluate PDA size and mean pulmonary artery pressure on QTc, QTd, and PR interval before PDA closure.

## 3 RESULTS

As shown in Table 1, we included 97 children (48 males and 49 females), with a mean age of 31.36 ± 4.26 months and mean weight of 12.44 ± 7.46 kg, into two groups of High PAP (30 patients) and normal PAP (67 patients), among which 52 patients (53%) had a small size, 38 patients (37%) had a moderate size, and 7 patients (10%) had a large size PDA. Sixty patients (61.9%) had a normal cardio/thoracic ratio, and 37 patients (38.1%) had an increased C/T ratio. Sixty‐five patients (67.0%) had normal pulmonary blood flow, while 32 patients (33.0%) had increased PBF. Mean PAP in patients with moderate to large PDA was 22.48±7.56 mm Hg which was in contrast with the mean PAP in small PDA group 18.44±5.72 mm Hg (*p *= .0001). In order to adjust the relationship between mean PAP and PDA size, meta‐regression logistics analysis was used to assess the relationship between PDA size and electrocardiographic variables. Analysis showed statistically significant correlation between PDA size and QTc (*p *= .034) and also PDA size and QTd (*p *= .049), compared to PDA size and PR interval which was not statistically significant (*p*=0.544). Due to the non‐normal distribution of mean PAP data, inverse of square of mean PAP was used for regression analysis that showed there was no significant relationship between mean PAP and QTc, QTd, and PR interval before PDA closure (*p* = .40, *p* = .15, and *p* = .30, respectively).

**TABLE 1:** Demographic data and related *p*‐values in different groups (Total, normal, and high PAP)

Variable	Total (97)	Normal PAP (67)	High PAP (30)	*p*‐value
Age (month, mean,SE)	31.36 (3.48)	33.22 (4.24)	27.23 (6.13)	.43
Weight (kg, mean,SE)	12.45 (0.76)	13.01 (0.94)	11.2 (1.25)	.27
Sex (male, %)	48 (49.5%)	37 (55.2%)	11 (36.6%)	.09
Size of PDA (small, %)	52 (53.6%)	51 (76.1%)	1 (3.4%)	< .0001
C/T ratio (normal, %)	60 (61.9%)	58 (86.6%)	2 (6.7%)	< .0001
PBF (normal, %)	65 (67%)	63 (94%)	2 (6.7%)	< .0001

As shown in Table 2, the mean PR interval before PDA closure was 144.95 ± 5.28 msec, and the mean PR interval after the intervention was 142.84 ± 4.67 msec. There was significant change between before and after intervention PR interval (*p *< .0001).

**TABLE 2:** Electrocardiographic variables and related *p*‐values in different groups (Total, normal, and high PAP)

Variable	Total (97)		Normal PAP (67)		High PAP (30)		1st *p*‐value	2nd *p*‐value	3rd *p*‐value	4th *p*‐value
	Before msec ± SD	After msec ± SD	Before msec ± SD	After msec ± SD	Before msec ± SD	After msec ± SD				
QTc (ms)	407.03 ± 17.61	399.07 ± 15.31	403.99 ± 17.98	398.54 ± 16.11	413.83 ± 14.88	400.83 ± 13.52	<.0001	=.0001	<.0001	.0002
QT dispersion (ms)	34.72 ± 5.89	30.47 ± 4.38	32.90 ± 5.41	30.30 ± 4.69	38.80 ± 4.83	30.87 ± 3.61	<.0001	<.0001	<.0001	<.0001
PR interval (ms)	144.95 ± 5.28	142.84 ± 4.67	144.55 ± 5.28	143.36 ± 4.80	145.83 ± 5.27	141.67 ± 4.22	<.0001	.002	< .0001	.0001

Note

1st value: between before & after in total group. 2nd value: between before & after in normal PAP group. 3rd value: between before & after in high PAP group. 4th value: between normal & high PAP group.

**FIGURE 1:**
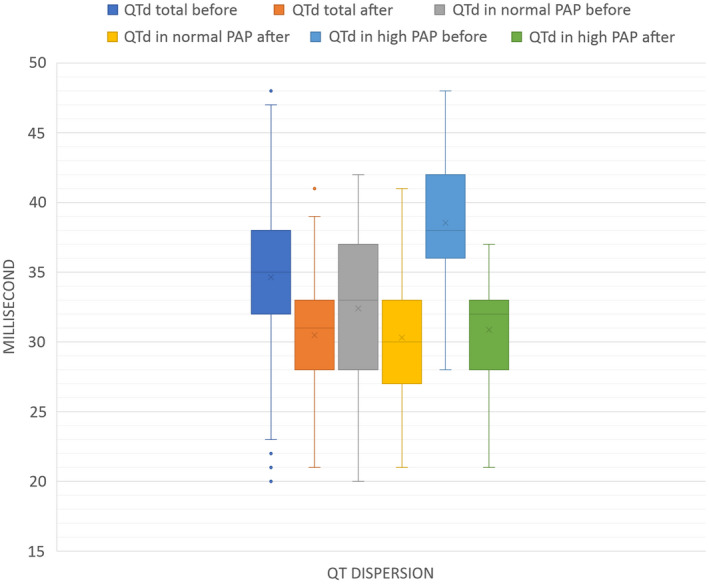
Descriptive values of QT dispersion in different groups. Time is shown in milliseconds (msec) on the vertical axis (*y*). QT dispersions (QTd) in different groups of our study are shown on the horizontal axis (*x*). These groups are in order from left to right: QTd in the general group before the intervention (PDA device closure) in light blue, QTd in the general group after the intervention in orange. QTd in normal PAP group before the intervention in gray and QTd in normal PAP group after intervention in yellow. QTd in high PAP group before the intervention in dark blue and finally QTd in high PAP group after the intervention in green. The two bars on the left in blue and orange show the QTd changes in all patients (general group). The mean, median, and maximum QTd levels decreased after the intervention, while in the statistical analysis, the decrease in QTd in the general group was significant (*p *< .0001). QTd changes in group A (normal PAP) are shown in two middle bars in gray and yellow. In this group, the mean, median, and maximum QTd levels decreased after the intervention, which were significant in statistical analysis (*p *= < 0.0001). The bars on the right show the QTd changes in group B (high PAP) in blue and green. All parameters, including median, mean, the minimum, and maximum QTd decreased after the intervention, which was a significant change (*p *< .0001)

As shown in Figure 1, Mean QTd before the procedure was 34.72±5.89 msec, and mean QTd after the intervention was 30.47±4.38 msec, and there was significant decrease in QTd 3 months after PDA closure in the general group (*p*<0.0001).

**FIGURE 2:**
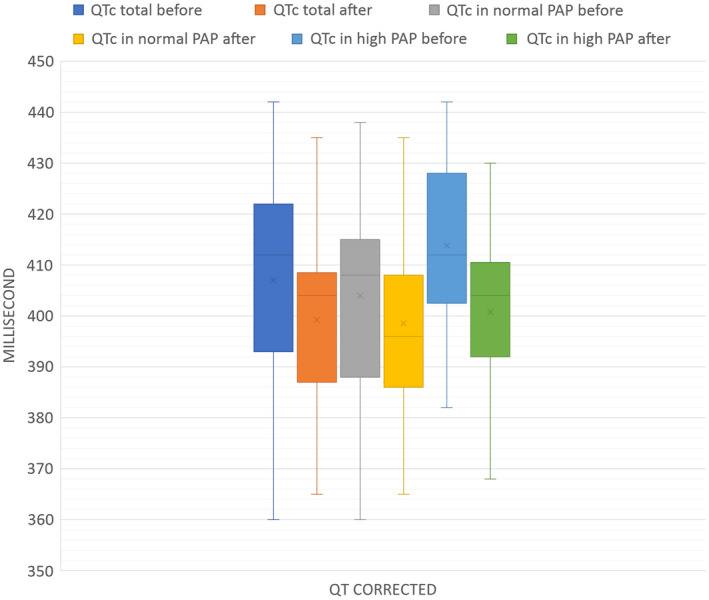
Descriptive values of QT corrected in different groups. Time is shown in milliseconds (msec) on the vertical axis (*y*). QT corrected (QTc) in different groups of our study are shown on the horizontal axis (*x*). These groups are in order from left to right: QTc in the general group before the intervention (PDA device closure) in light blue, QTc in the general group after the intervention in orange. QTc in normal PAP group before the intervention in gray and QTc in normal PAP group after intervention in yellow. QTc in high PAP group before the intervention in dark blue and finally QTc in high PAP group after the intervention in green. The two bars on the left in blue and orange show the QTc changes in all study patients. The mean, median, and maximum QTc decreased after the intervention and these changes were significant in the general group (*p *< .0001). QTc changes in group A (normal PAP) are shown in two middle bars in gray and yellow. In this group, the mean and maximum QTc levels decreased after the intervention, but these changes were not significant in statistical analysis (*p *= .0001). The bars on the right show the QTc changes in group B (high PAP) in blue and green. The mean, median, the minimum and maximum QTc decreased after the intervention, which was a significant change (*p *< .0001)

As shown in Figure 2, QTc dropped significantly after 3 months of PDA device closure at an average of 7.96 msec (*p*<0.0001). Mean QTc before and after the intervention was 407.03 msec, 399.07 msec, respectively.

In the normal pulmonary arterial pressure group (MPA=<20) labeled Group A (67 patients), the reduction in all the three variables QTc, QTd, and PR interval was significant (*p*=0.0001, *p*<0.0001, and *p*=0.002, respectively).

However, in Group high pulmonary arterial pressure (MPA>20 mmHg), QTc and QTd after 3 months of PDA device closure significantly decreased (*p* < .0001, *p* < .0001, respectively), and PR interval significantly increased (*p* < .0001).

It is noteworthy that the coil type device was used in 59 patients, while the Amplatzer PDA Occluder device was used in 38 patients. Furthermore, none of the patients had any residual PDA in post‐intervention echocardiography.

## 4 DISCUSSION

Ventricular repolarization characteristics measured by QT interval play a significant role in arrhythmogenesis (Merx et al., 1977). Dispersion of repolarization, which reflects regional heterogeneity of the recovery process in the myocardium, is imperative to the genesis of ventricular arrhythmias (Higham et al., 1994). This spatial heterogeneity is assessed by multiple indicators, including QT dispersion, T‐wave peak‐to‐end interval, beat‐to‐beat variability of T wave morphology, etc. According to previous studies, the predictive value of QT dispersion, defined as the difference between longest and shortest QT interval among all 12 leads of a standard surface ECG, has been under question (Malik & Batchvarov, 2000), but a recent meta‐analysis of available studies has confirmed that greater QT dispersion is significantly associated with ventricular arrhythmias (Bazoukis et al., 2020). Besides, we know that angiography is one of the modalities in PDA closure in pediatrics. Few studies have scrutinized the value of QTd and QTc in pediatrics, particularly regarding the PDA closure. This study aimed to fill in the gap and examine the QTc, QTd, and PR interval changes following the angiographic intervention of PDA device closure.

QT and QTd have a circadian rhythm (Kula et al., 2004); therefore, QTd measurements should be made at the same of day, and we assessed QTc and QTd in the morning time between 10 to 12 o'clock. Current study population had a mean QTc of 407.03 ± 17.61, which is in normal range (from 350–360 to 450–460 ms) (Postema & Wilde, 2014) and their mean QT dispersion was 34.72 ± 5.89 ms. Normal subjects had a mean QTd value of 33 ms (range 10–71 ms) (Malik & Batchvarov, 2000); QTd values >80 ms were shown to increase the risk of cardiovascular mortality by four‐fold when compared with subjects with QTd values < 30 ms (Elming et al., 1998); Patients with long QT syndrome who showed values in excess of QTd >100 ms were at high risk (Priori et al., 1994). In our study show that QTd is almost in normal range. We do not have any patient with QTd above 50 ms.

Considering the effect of PAH on the prolongation of QTc, QTd, and PR interval in previous studies, we also examined its role on the three variables before and after the intervention. According to the Sixth Pulmonary Hypertension World Symposium 2018, PAH refers to the mean pulmonary artery pressure above 20 at rest. Some studies showed that QTd and QTc increase after open‐heart surgery compared to the pre‐operation period (Alp et al., 2012). Nonetheless, our statistical studies showed that QTc significantly decreases after 3 months of PDA device closure. It seems that in the general group, left‐to‐right shunt correction decreases the depolarization time of the ventricles (QT interval).

PR interval prolongation has been associated with adverse outcomes, such as increasing risk of atrial fibrillation (Cheng et al., 2009). In patients with pulmonary hypertension, there is also some evidence of sinus and atrioventricular node dysfunction (Temple, 2017) and first‐degree AV block (Cirulis et al., 2019). In our study, as shown in Table 2, PDA closure had the effect on PR interval, but in the children with PDA and pulmonary hypertension, PR interval significantly reduced in comparison with patient with PDA and normal PAP (*p*‐value=.0001).

PDA can affect the hemodynamics, and hemodynamics may affect ventricular repolarization. But the effect seems to be quite small, In PDA patients, there should be RV pressure overload and PA/PV/LA/LV volume overload. Therefore, ventricular repolarization abnormality should be mixture of both. And if there is some increase in PA resistance, the hemodynamic situation will be quite different.

Furthermore, difference between QTc, QTd, and PR interval changes in normal and high PAP group was significant (*p*‐value = .0002, *p*‐value < .0001, *p*‐value = .0001, respectively) 3 months after the procedure. In other words, pulmonary arterial hypertension has more powerful effect on heterogeneity of ventricular depolarization, ventricular depolarization duration, and atrial‐ventricular depolarization interval than overflow shunt caused by PDA. According to previous studies, we realized that the reduced QTd after PDA device closure might reduce cardiac complications, such as arrhythmia and sudden death after the intervention especially in children with pulmonary hypertension.

## 5 CONCLUSION

QTc and QTd significantly decrease 3 months after PDA device closure. In two groups normal and high PAP, after PDA device closure significantly reduces QTc and QTd. So, in patients with high pulmonary arterial pressure, QTc and QTd decrease significantly after 3 months of intervention. Pulmonary arterial pressure seems to significantly affect QTd elevation especially with left‐to‐right shunt caused by PDA. However, a left‐to‐right shunt through the patent ductus arteriosus can affect ventricular repolarization; this effect seems to be particularly more significant when there is pulmonary hypertension.

### 5.1 Limitation

There is a great heterogeneity in the measurement methods of ECG markers and still there is no consensus on the best approach for assessing ECG markers. There is some source of heterogeneity in measuring ECG intervals that contain the T wave such as: Presence of the U wave in right precordial leads, extracorporeal noises, body movements, and inter‐operator variabilities.

## CONFLICT OF INTEREST

The authors declare no conflicts of interest.

## AUTHOR CONTRIBUTIONS

Conception and design of the research: MR Khalilian; Acquisition of data: MR Khalilian and M Ziaratban; Analysis and interpretation of the data: MR Khalilian and A Shirvani; Writing of the manuscript: MR khalilian, M Ziaratban, P Alizadeh, and AR Norouzi; Critical revision of the manuscript for intellectual content: MR Khalilian and M Ziaratban. All authors read and approved the final draft.

## ETHICAL APPROVAL

No funding was received for this study. The Ethical Committee of the Faculty of Medicine approved the study (Ethical number: IR.SBMU.MSP.REC.1400.125).

## Data Availability

The data that support the findings of this study are available from the corresponding author upon reasonable request.

## References

[anec12945-bib-0001] Alp, H. , Narin, C. , Tamer, B. , & Sarıgül, A. (2012). Evaluation of pre‐and postoperative corrected QT dispersion predicting the development of arrhythmias in children undergoing congenital heart surgery. Turkish Journal of Thoracic and Cardiovascular Surgery, 20(4), 716–721.

[anec12945-bib-0002] Barr, C. S. , Naas, A. , Freeman, M. , Lang, C. C. , & Struthers, A. D. (1994). QT dispersion and sudden unexpected death in chronic heart failure. The Lancet, 343(8893), 327–329. 10.1016/S0140-6736(94)91164-9 7905146

[anec12945-bib-0003] Bazoukis, G. , Yeung, C. , Wui Hang Ho, R. , Varrias, D. , Papadatos, S. , Lee, S. , Ho Christien Li, K. , Sakellaropoulou, A. , Saplaouras, A. , Kitsoulis, P. , Vlachos, K. , Lampropoulos, K. , Thomopoulos, C. , Letsas, K. P. , Liu, T. , & Tse, G. (2020). Association of QT dispersion with mortality and arrhythmic events—A meta‐analysis of observational studies. J Arrhythmia, 36, 105–115. 10.1002/joa3.12253 PMC7011802 32071628

[anec12945-bib-0004] Blužaitè, I. , Braždžionytè, J. , Žaliūnas, R. , Rickli, H. , & Ammann, P. (2006). QT dispersion and heart rate variability in sudden death risk stratification in patients with ischemic heart disease. Medicina (Kaunas), 42(6), 450–454. 16816538

[anec12945-bib-0005] Brendorp, B. , Elming, H. , Jun, L. , Køber, L. , Malik, M. , Jensen, G. B. & Torp‐Pedersen, C. (2001). QT dispersion has no prognostic information for patients with advanced congestive heart failure and reduced left ventricular systolic function. Circulation, 103(6), 831–835. 10.1161/01.CIR.103.6.831 11171791

[anec12945-bib-0006] Cheng, S. , Keyes, M. J. , Larson, M. G. , McCabe, E. L. , Newton‐Cheh, C. , Levy, D. , Benjamin, E. J. , Vasan, R. S. , & Wang, T. J. (2009). Long‐term outcomes in individuals with prolonged PR interval or first‐degree atrioventricular block. JAMA, 301(24), 2571–2577. 10.1001/jama.2009.888 19549974 PMC2765917

[anec12945-bib-0007] Cirulis, M. M. , Ryan, J. J. , & Archer, S. L. (2019). Pathophysiology, incidence, management, and consequences of cardiac arrhythmia in pulmonary arterial hypertension and chronic thromboembolic pulmonary hypertension. Pulmonary Circulation, 9(1), 1–15. 10.1177/2045894019834890 PMC6410395 30747032

[anec12945-bib-0008] Day, C. P. , McComb, J. M. , & Campbell, R. (1990). QT dispersion: An indication of arrhythmia risk in patients with long QT intervals. Heart, 63(6), 342–344. 10.1136/hrt.63.6.342 PMC1024518 2375895

[anec12945-bib-0009] Ece, İ. , Üner, A. , Ballı, Ş. , Oflaz, M. B. , Kibar, A. E. , & Sal, E. (2014). P‐wave and QT interval dispersion analysis in children with Eisenmenger syndrome. Turk Kardiyoloji Dernegi Arsivi: Turk Kardiyoloji Derneginin Yayin Organidir, 42(2), 154–160. 10.5543/tkda.2014.68704 24643147

[anec12945-bib-0010] Elming, H. , Holm, E. , Jun, L. , Torp‐Pedersen, C. , Køber, L. , Kircshoff, M. , Malik, M. , & Camm, J. (1998). The prognostic value of the QT interval and QT interval dispersion in all‐cause and cardiac mortality and morbidity in a population of Danish citizens. European Heart Journal, 19(9), 1391–1400. 10.1053/euhj.1998.1094 9792266

[anec12945-bib-0011] Farag, M. , El Amrousy, D. , El‐Serogy, H. , & Zoair, A. (2018). Role of plasma asymmetric dimethyl‐L‐arginine levels in detection of pulmonary hypertension in children with CHD. Cardiology in the Young, 28(9), 1163–1168. 10.1017/S1047951118001026 29950194

[anec12945-bib-0012] Fei, L. , Goldman, J. , Prasad, K. , Keeling, P. , Reardon, K. , Camm, A. et al (1996). QT dispersion and RR variations on 12‐lead ECGs in patients with congestive heart failure secondary to idiopathic dilated cardiomyopathy. European Heart Journal, 17(2), 258–263. 10.1093/oxfordjournals.eurheartj.a014843 8732380

[anec12945-bib-0013] Higham, P. , & Campbell, R. (1994). QT dispersion. British Heart Journal, 71(6), 508–510. 8043327 10.1136/hrt.71.6.508 PMC1025441

[anec12945-bib-0014] Kula, S. , Olgunturk, R. , Tunaoglu, F. S. , & Canter, B. (2004). Circadian variation of QTc dispersion in children with vasovagal syncope. International Journal of Cardiology, 97(3), 407–410. 10.1016/j.ijcard.2003.10.024 15561326

[anec12945-bib-0015] Linker, N. J. , Colonna, P. , Kekwick, C. A. , Till, J. , Camm, A. J. , & Ward, D. E. (1992). Assessment of QT dispersion in symptomatic patients with congenital long QT syndromes. The American Journal of Cardiology, 69(6), 634–638. 10.1016/0002-9149(92)90155-R 1346947

[anec12945-bib-0016] Malik, M. , & Batchvarov, V. N. (2000). Measurement, interpretation and clinical potential of QT dispersion. Journal of the American College of Cardiology, 36(6), 1749–1766. 10.1016/S0735-1097(00)00962-1 11092641

[anec12945-bib-0017] Merx, W. , Yoon, M. S. , & Han, J. (1977). The role of local disparity in conduction and recovery time on ventricular vulnerability to fibrillation. American Heart Journal, 94(5), 603–610. 10.1016/S0002-8703(77)80130-0 910699

[anec12945-bib-0018] Mirvis, D. M. (1985). Spatial variation of QT intervals in normal persons and patients with acute myocardial infarction. Elsevier. 10.1016/s0735-1097(85)80387-9 3973259

[anec12945-bib-0019] Moreno, F. L. , Villanueva, T. , Karagounis, L. A. , & Anderson, J. L. (1994). Reduction in QT interval dispersion by successful thrombolytic therapy in acute myocardial infarction. TEAM‐2 Study Investigators. Circulation, 90(1), 94–100. 10.1161/01.CIR.90.1.94 8026057

[anec12945-bib-0020] Naas, A. A. , Davidson, N. C. , Thompson, C. , Cummings, F. , Ogston, S. A. , Jung, R. T. , Newton, R. W , & Struthers, A. D. (1998). QT and QTc dispersion are accurate predictors of cardiac death in newly diagnosed non‐insulin dependent diabetes: Cohort study. BMJ, 316(7133), 745–746. 10.1136/bmj.316.7133.745 9529410 PMC28479

[anec12945-bib-0021] Postema, P. G. , & Wilde, A. A. (2014). The measurement of the QT interval. Current Cardiology Reviews, 10(3), 287–294. 10.2174/1573403x10666140514103612 24827793 PMC4040880

[anec12945-bib-0022] Priori, S. G. , Napolitano, C. , Diehl, L. , & Schwartz, P. J. (1994). Dispersion of the QT interval. A marker of therapeutic efficacy in the idiopathic long QT syndrome. Circulation, 89(4), 1681–1689. 10.1161/01.CIR.89.4.1681 7908611

[anec12945-bib-0023] Pye, M. , Quinn, A. , & Cobbe, S. (1994). QT interval dispersion: a non‐invasive marker of susceptibility to arrhythmia in patients with sustained ventricular arrhythmias? Heart, 71(6), 511–514. 10.1136/hrt.71.6.511 PMC1025443 8043329

[anec12945-bib-0024] Rajdev, A. , Garan, H. , & Biviano, A. (2012). Arrhythmias in pulmonary arterial hypertension. Progress in Cardiovascular Diseases, 55(2), 180–186. 10.1016/j.pcad.2012.06.002 23009914 PMC3832144

[anec12945-bib-0025] Saleh, A. , Shabana, A. , El Amrousy, D. , & Zoair, A. (2019). Predictive value of P‐wave and QT interval dispersion in children with congenital heart disease and pulmonary arterial hypertension for the occurrence of arrhythmias. Journal of the Saudi Heart Association, 31(2), 57–63. 10.1016/j.jsha.2018.11.006 30618481 PMC6312787

[anec12945-bib-0027] Temple, I. P. (2017). Arrhythmias in pulmonary arterial hypertension. Journal of Congenital Cardiology, 1(1), 1–6. 10.1186/s40949-017-0004-8

[anec12945-bib-0028] Zaidi, M. (1996). Dispersion of ventricular repolarization in hypertrophic cardiomyopathy. Journal of Electrocardiology, 29, 89–94. Université catholique de Louvain. 9238384 10.1016/s0022-0736(96)80026-4

